# Increases in diagnosis and management of obstetric and neonatal complications in district hospitals during a high intensity nurse-mentoring program in Bihar, India

**DOI:** 10.1371/journal.pone.0247260

**Published:** 2021-03-18

**Authors:** Ammar Joudeh, Rakesh Ghosh, Hilary Spindler, Seema Handu, Sunil Sonthalia, Aritra Das, Aboli Gore, Tanmay Mahapatra, Dilys Walker

**Affiliations:** 1 Department of Obstetrics, Gynecology, and Reproductive Sciences, University of California San Diego, San Diego, California, United States of America; 2 Institute for Global Health Sciences, University of California, San Francisco, California, United States of America; 3 PRONTO International, State RMNCH+A, Patna, India; 4 CARE India, Patna, Bihar, India; 5 School of Medicine and Department of Obstetrics-Gynecology and Reproductive Sciences, University of California San Francisco, San Francisco, California, United States of America; ESIC Medical College & PGIMSR, INDIA

## Abstract

Maternal and neonatal mortality in Bihar, India was far higher than the aspirational levels set out by the Sustainable Development Goals. Provider training programs have been implemented in many low-resource settings to improve obstetric and neonatal outcomes. This longitudinal investigation assessed diagnoses and management of postpartum hemorrhage (PPH), hypertensive disorders of pregnancy, birth asphyxia (BA), and low birth weight (LBW), as part of the CARE’s AMANAT program in 22 District Hospitals in Bihar, between 2015 and 2017. Physicians and nurse mentors conducted clinical instruction, simulations and teamwork and communication activities, infrastructure and management support, and data collection for 6 consecutive months. Analysis of diagnosis included 11,259 non-referred and management included 11,800 total (non-referred and referred) admissions that were observed. Data were analyzed using the chi-square test for trend. PPH was diagnosed in 3.7% with no significant trend but diagnosis of hypertensive disorders increased from 1.0% to 1.7%, (p_trend_ = 0.04), over the 6 months. BA was diagnosed in 5.8% with no significant trend but LBW diagnoses increased from 11% to 16% (p_trend_<0.01). Among PPH patients, 96% received fluids, 85% received uterotonics and 11% received Tranexamic Acid (TXA). There was a significant positive trend in the number of patients receiving TXA for PPH (6% to 13.8%, p_trend_ = 0.03). Of all neonates with BA, there were statistically significant increases in the proportion who were initially warmed, dried, and stimulated (78% to 94%, p_trend_ = 0.02), received airway suction (80% to 93%, p_trend_ = 0.03), and supplemental oxygen without positive pressure ventilation (73% to 86%, p_trend_ = 0.05). Diagnoses of hypertensive disorders and LBW as well as initial management of BA increased during the AMANAT program. However, underdiagnoses of PPH and hypertensive disorders relative to population levels remain critical barriers to improving maternal morbidity and mortality.

## Introduction

Globally, there were about 275,000 maternal deaths and 2.7 million neonatal deaths in 2015 [1,2]. Despite increasing institutional deliveries from 28% to 64% between 2007 and 2016, the concurrent maternal mortality ratio (MMR) in Bihar, India was 165 per 100,000 live births from 2014–2016, and the neonatal mortality rate (NMR) was 27 per 1,000 live births [3]. These figures are among the highest in India, and far above the aspirational Sustainable Development Goal of less than 70 per 100,000 live births for MMR and 12 per 1,000 live births for NMR [4–6]. Postpartum hemorrhage (PPH) and hypertensive disorders of pregnancy (HDP), each impact 5–10% of deliveries [7,8], and Birth asphyxia (BA) impacts 5–10% of neonates. These are leading causes of mortality. Low birth weight (LBW) incidence varies widely by context but is estimated as 20–30% of all births in India, and its diagnosis can lead to life-saving interventions for the newborn [9].

Efforts to improve care during obstetric and neonatal emergencies have focused on improving rates of institutional deliveries and the quality of care [10]. However, recent evidence in India and globally suggests that increases in institutional deliveries and reported availability of treatment have not tracked improvements in outcomes [11,12]. Reducing mortality goes beyond availability of treatment; it also depends on early recognition of serious maternal and neonatal complications, so that appropriate treatment can be provided timely [13,14].

One approach to improving timely diagnosis and treatment of complications is to provide simulation based team training and bedside mentoring [15,16]. Mentoring and simulation trainings have shown promise in improving quality of obstetric and neonatal care in emergencies, though evidence remains limited. A randomized control trial of a coaching-based implementation of the World Health Organization’s Safe Childbirth Checklist in Uttar Pradesh, India showed increases in adherence of several practices, but no changes in mortality [17]. The Helping Babies Breathe intervention, which aimed to use simulation to improve neonatal resuscitation (NR) practices, has shown to reduce fresh stillbirths in India and Tanzania and NMR in Tanzania [18,19]. PRONTO’s simulation and team training program has been shown to improve knowledge, self-efficacy, and facility processes in Mexico and Guatemala. Evidence suggests PRONTO training led to a transient decrease in neonatal mortality in Mexican hospitals [20,21]. While these interventions focus on both recognition and treatment of complications, their evaluations have not specifically examined whether complication diagnoses were commensurate to expected rates.

In Bihar, CARE India, a non-governmental organization, collaborated with the State Government of Bihar to implement a nurse-mentoring program called *Apatkaleen Matritva evam Navjat Tatparta* (AMANAT)–Vyapak, or “Comprehensive Emergency Obstetric and Neonatal Readiness” in all 38 districts of the state between 2015 and 2017. The goal of the AMANAT nurse mentoring program is to reduce maternal and neonatal deaths by improving facility-based intrapartum obstetric and neonatal care. The program integrated PRONTO’s simulation and team training, bedside mentoring, demonstrations using mannequins, didactic lessons, and logistics support. This program was unique in its intensity, attaching nurse mentors for 6 consecutive months, one each in 22 district hospitals (DH), with a focus on diagnosis, management, and teamwork and communication (T&C) between providers that are necessary to successfully diagnose and transition to appropriate management. The same AMANAT nurse-mentoring program in lower level BEMONC facilities, was shown to improve provider capacity to identify PPH and BA cases [22]. This longitudinal investigation aims to assess: 1) whether there are changes in diagnosis of PPH, HDP, BA and LBW; and 2) whether there are changes in the frequency with which key treatment steps of these complications are performed over the course of the AMANAT-CEMONC (Comprehensive Emergency Obstetric and Neonatal Care) intervention.

## Materials and methods

### Study setting and participants

DHs in Bihar, India are referral centers, each serves a population of approximately 3 million population and conducts several hundred deliveries per month [23]. Pregnant women may present directly to the DHs or be referred from a lower level facility in the event of a complication. Obstetric and neonatal care at DHs in Bihar is primarily provided by nurse midwives. Physicians are on call but are often not available during births and must be called to the facility when assistance is needed. We followed the SQUIRE guidelines for reporting quality improvement programs, as recommended by the EQUATOR network [24].

### Intervention

The AMANAT was a quality improvement program implemented by CARE India in collaboration with the Government of Bihar, in which a nurse-mentoring model was used to improve maternal and neonatal health outcomes. AMANAT mentors are nurse midwives who were trained by CARE India, in collaboration with PRONTO International, to provide simulation training, T&C activities, structured debriefing, bedside clinical mentoring, demonstration of procedures, and didactic lectures. Mentees in CEMONC DHs were Auxiliary Nurse Midwives (ANM) or General Nurse Midwives (GNM) with 2 or 3.5 years of training after grade 12 education, respectively. This manuscript deals with the AMANAT Vyapak version of the program that was implemented in 22 DHs of Bihar.

PRONTO International’s simulation and team training curriculum was tailored to the Bihar context and emphasized diagnosis and management of common obstetric and neonatal complications, T&C techniques, and person-centered maternity care. The simulation scenarios were integrated into the AMANAT program and focused on normal spontaneous vaginal delivery (NSVD), post-partum hemorrhage (PPH), preeclampsia (PE), and NR. Simulations were recorded on video, and video-aided debriefs were conducted after every simulation to identify areas of improvement and build a culture of safe learning space with open communication. T&C training was based on the TEAMSTEPPS^™^ approach and included structured team-building activities and training on specific communication techniques [25].

AMANAT mentors spent 5 days a week for 6 consecutive months in each of 22 DHs. In addition to simulation and team training, AMANAT mentors provided clinical mentoring during live births. The broader quality improvement program implemented by CARE India also strengthened infrastructure and management, infection control, and hazardous waste disposal.

### Data collection

Data collection systems for the AMANAT program were developed and managed by CARE-India in Patna. During the intervention between 2015 and 2017, AMANAT mentors collected data on all training activities, as well as maternal and neonatal clinical data, while they were in their assigned facilities carrying out mentoring activities. Data was collected and entered by the mentors in a web-based Facility Information System (FIS) dataset, daily. Data from periods when mentors were not in-house at night were extracted from facility registers and cross-checked with the staff the following day. Data included patient demographics, mode of delivery, complication diagnoses, and management. Raw data was cleaned and checked by trained staff at the CARE-India office in Patna and patient identifiable data were removed. De-identified data was used for analysis.

### Outcome definitions

Standard WHO definitions were used to the extent possible, in the study setting. For PPH and NR, definitions were modified due to operational constraints, as described previously [22]. For PPH, due to inconsistent availability of equipment necessary to quantitatively determine blood loss, the operational definition of PPH was, “a provider observing persistent trickling of more than expected blood loss, or a blood clot that was the size of a fist or changing pads every 5–15 minutes.” For BA, the standard WHO definition for BA was used: failure to initiate or sustain breathing at birth. However, to instill urgency, the algorithm was modified from ILCOR guidelines to recommend initiation of PPV in the first 30 seconds, rather than 1 minute. In this study, HDP includes, pregnancy-induced hypertension, preeclampsia, and eclampsia. We lacked data on chronic hypertension [8].

### Statistical analysis

This was an interventional study with no control group. We estimated the proportion of complicated deliveries, longitudinally over time. The incidences of maternal and neonatal complications recorded in the FIS were estimated as proportions of all deliveries and all births, respectively. Also, the proportion of direct admissions to the DHs, those referred-in from a lower-level facility, the number of admissions that were directly observed and those that were added to the dataset from after-hours facility registers were estimated. Trends in PPH, HDP, BA, and LBW diagnosis by month of intervention were examined for direct non-referred admissions that were observed by AMANAT mentors. As the outcomes were proportions, we used Pearson’s chi-square test of trend over time (i.e., 6 months). Trend analysis was performed for direct admissions only, as referred admissions are likely to arrive with a suspected diagnosis, including them would not assess the independent ability of providers to diagnose complications. The above complications were chosen because they were the focus of the training intervention, as key contributors to maternal and neonatal mortality. Rates of additional complications are presented in the supplement.

We also analyzed trends by month of intervention in the completion of key steps of the management of PPH and BA cases because adequate data on management of only these two complications were available. In PPH cases, these steps included administration of fluids, uterotonics, and tranexamic acid (TXA), though TXA was only recorded consistently in the facilities that were included in the last phase of the intervention. We lacked data on invasive treatments for PPH such as uterine balloon tamponade and laparotomy. For BA, the following steps of the NR algorithm were analyzed: warming, drying, and stimulating the neonate, suctioning the airway, use of the radiant warmer, use of oxygen by cannula, and use of positive pressure ventilation (PPV). The clinical significance of NR steps must be interpreted with caution given we do not have data to determine whether provision of oxygen and/or PPV were indicated. For HDP, administration of antihypertensives was recommended to target a blood pressure of less than 160/110 mm of Hg. Seizure prophylaxis with magnesium sulfate was recommended for preeclampsia with severe features, consistent with WHO guidelines [26]. Because we did not have data on actual blood pressures, and the numbers of severe preeclampsia and eclampsia cases were small, we were not able to assess the clinical significance of trends in preeclampsia treatment. Direct and referred admissions were included in the analysis of treatment trends, as even referred admissions who arrive with a presumed diagnosis would still require key management steps at the DH. For NR, where initial resuscitation steps should have occurred at the referring institution, we also ran the analysis without referred admissions to ensure the robustness of that result. Statistical analyses were conducted using Stata 15.1 (Stata Corp. TX).

Ethical approval for this study was granted from the Committee on Human Research at UCSF (14–15446) and the Institutional Committee for Ethics and Review of the Indian Institute of Health Management Research. Consent was obtained from study participants before data collection. Patient identifiable information was not used in the analytical dataset.

## Results

Characteristics of the study population including overall complication proportions are presented in Table 1. A total of 12,307 admissions were included in the FIS dataset. Of these, 11,616 (94%) were direct (non-referred) admissions and 11,800 (96%) were observed by mentors, of which 11,259 were both direct and observed admissions. Average maternal age was 24.6 years (Standard Deviation, SD = 3.5). Physicians were consulted by phone in 514 cases (4%) and in-person in 1,841 cases (15%). There were 10,574 spontaneous vaginal deliveries (86%), 1,168 cesarean sections (10%) and 18 operative vaginal deliveries (<1%). The remaining 547 (4%) of admissions were antenatal, postnatal, or referred to a higher-level facility before delivery. There were 9 maternal deaths and 10 neonatal deaths. Specific complications by month are shown in S1 Table.

**Table 1:** Admission characteristics and demographics in 22 Compressive Emergency Obstetric and Neonatal Care (CEMONC) facilities in Bihar, India during the AMANAT nurse-mentoring intervention.

	No.	%
**Type of admission**		
Direct	11,616	94.4
Referred	628	5.1
Unknown	63	0.5
**Delivery observation**		
Observed	11,800	95.9
Partially Observed	507	4.1
**Parity**		
0	4,214	34.2
1	3,894	31.6
2	2,446	19.9
3	1,147	9.3
4	392	3.2
5+	214	1.7
**Maternal age** (years), **median (IQR)**	24 (22–26)	
**Mode of delivery**		
Spontaneous vaginal delivery	10,574	85.9
Assisted vaginal delivery	18	0.2
Caesarian section	1,168	9.5
Unknown or not applicable	547	4.4
**Physician consulted**		
No consultation	9,952	80.9
Phone consultation	514	4.2
In-person consultation	1,841	15.0
**Aggregate complications**		
No maternal complication	8,792	71.4
Any maternal complication	3,515	28.6
No neonatal complication	10,069	81.8
Any neonatal complication	2,238	18.2
**Maternal deaths**	9	0.1
**Neonatal deaths**	10	0.1
**Total admissions**	**12,307**	

### Diagnosis and management of complications

**Fig 1:**
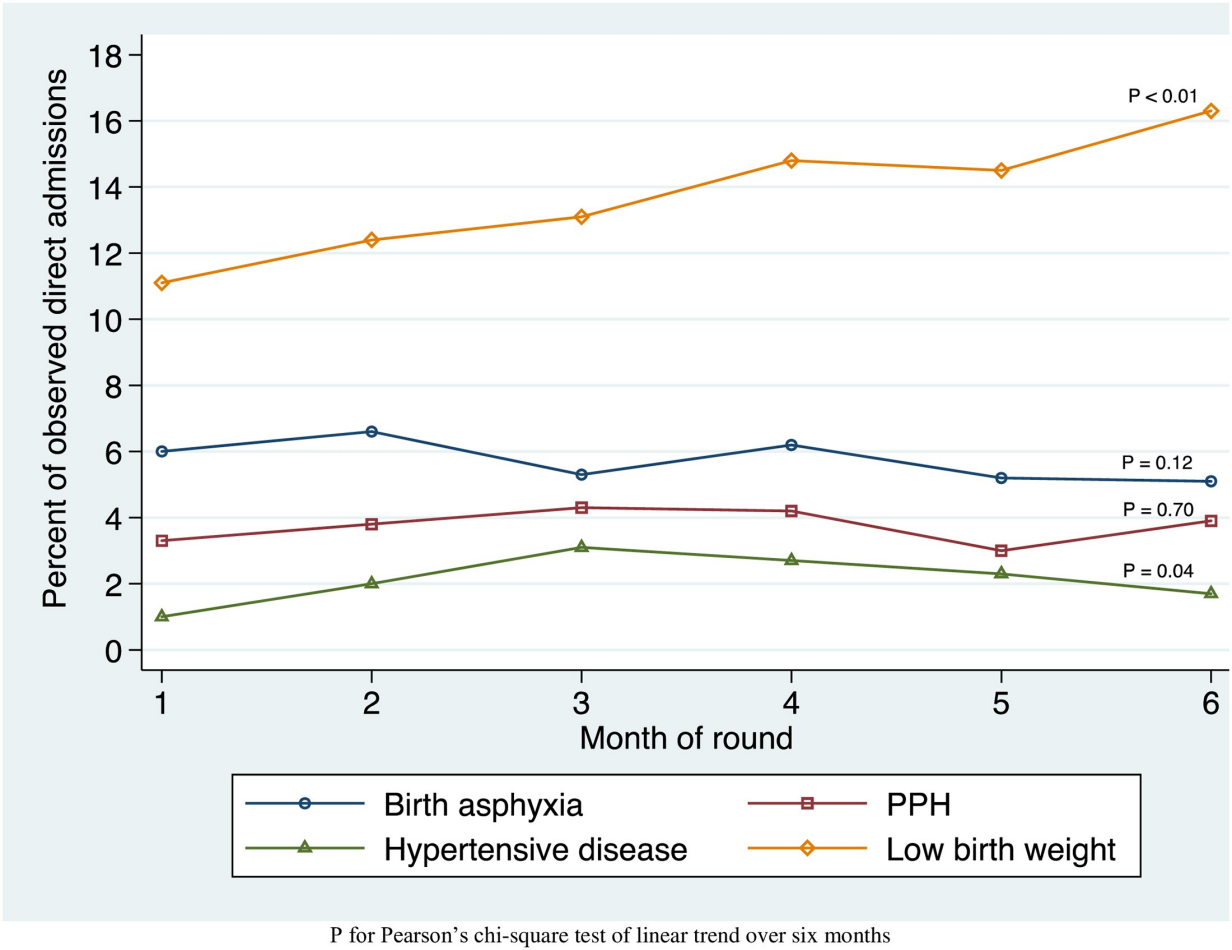
Trends in complication diagnosis by month in observed direct admissions to 22 CEMONC facilities in Bihar, India during the AMANAT nurse-mentoring intervention.

Trends in diagnosis of PPH, HDP, BA, and LBW are shown in Fig 1. Over the six months of the AMANAT intervention, in direct observed admissions, diagnosis of PPH increased from 3.3% to 3.9% of admissions (p_trend_ = 0.70). HDP increased from 1.0% to 1.7%, (p_trend_ = 0.04). BA decreased from 6.0% to 5.1% (p_trend_ = 0.12). LBW diagnoses increased from 11% to 16% (p_trend_<0.01). Other complications noted in the FIS dataset are shown in S1 Table.

**Fig 2:**
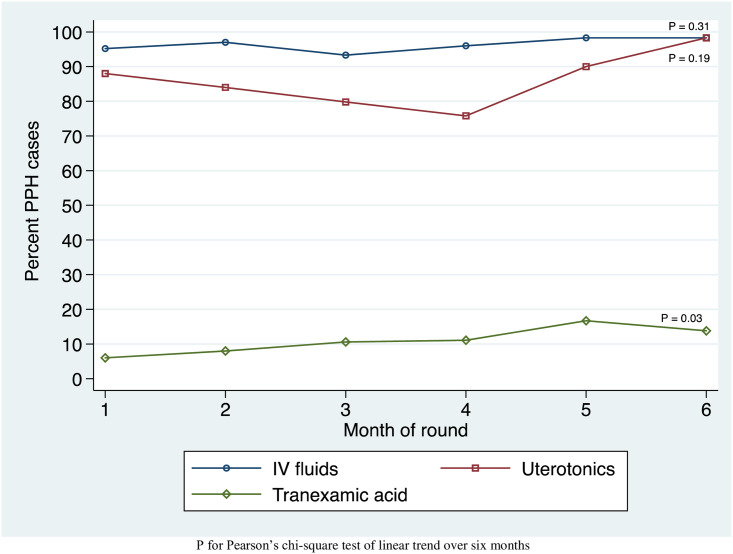
Trends in postpartum hemorrhage management by month in observed admissions to 22 CEMONC facilities in Bihar, India during the AMANAT intervention.

Trends in management of PPH in all observed direct and referred admissions are shown in Fig 2. Among 504 patients diagnosed with PPH in all observed admissions, use of fluids increased from 95.2% to 98.3% (p_trend_ = 0.31). Patients receiving uterotonics rose from 88.0% to 98.3% (p_trend_ = 0.19). Patients receiving TXA for PPH rose from 6% to 13.8% (p_trend_ = 0.03). Treatment steps for other complications are shown in S2 Table.

**Fig 3:**
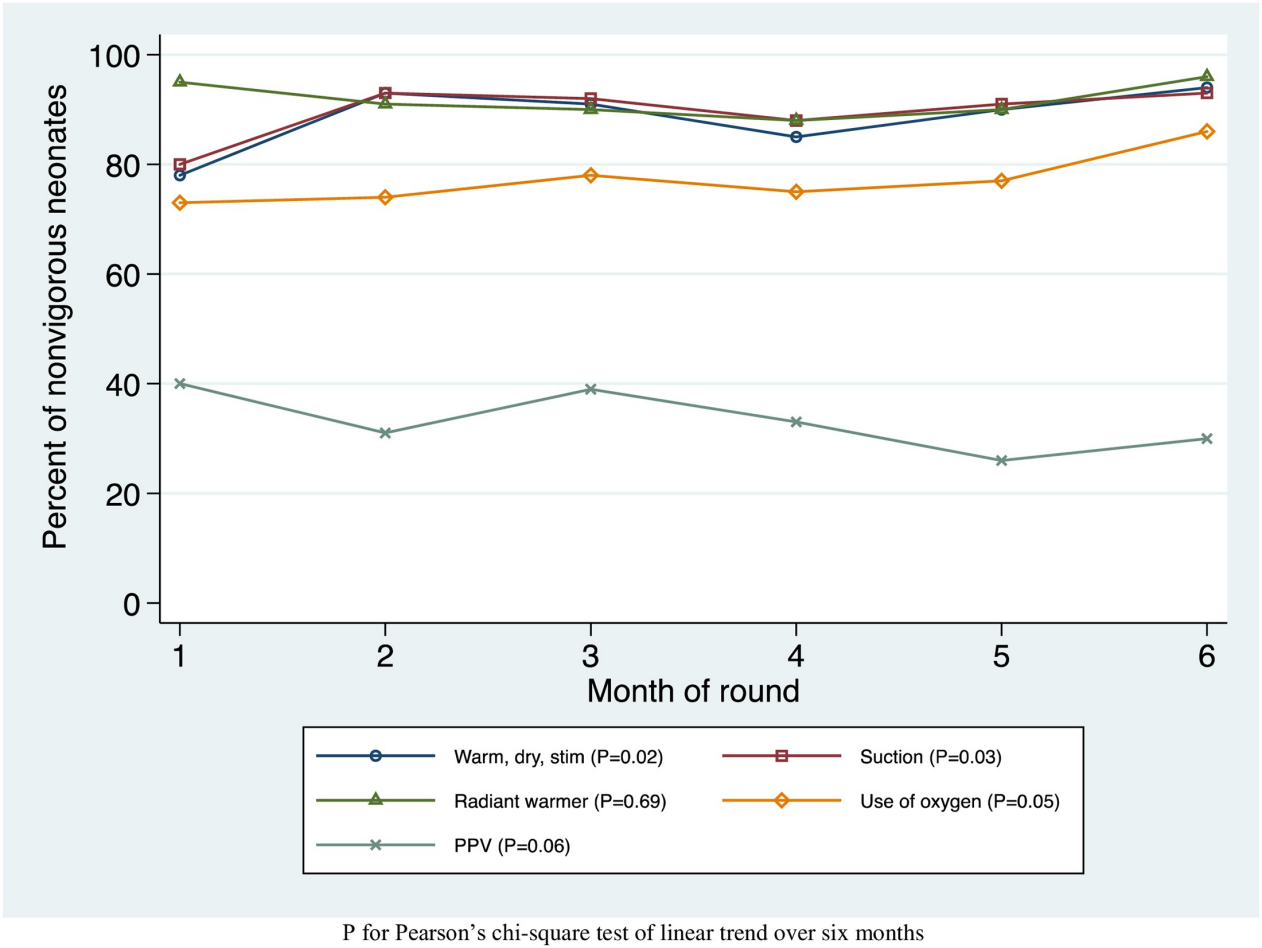
Trends in neonatal resuscitation by month in observed admissions to 22 CEMONC facilities in Bihar, India during the AMANAT nurse-mentoring intervention.

Trends in neonatal resuscitation steps are shown in Fig 3. There were 721 neonates with BA in all observed admissions, and 61 (8%) of these were born by cesarean section. Of all neonates with BA, there were statistically significant increases in the proportion who were initially warmed, dried, and stimulated (78% to 94%, p_trend_ = 0.02), received airway suction (80% to 93%, p_trend_ = 0.03), and received supplemental oxygen without PPV (73% to 86%, p_trend_ = 0.05). The increase in the proportion who were initially warmed, dried, and stimulated was robust to dropping referred admissions. The number of BA neonates receiving PPV appeared to have a decreasing trend, but this was not statistically significant (40% to 30%, p_trend_ = 0.06). The radiant warmer was used on 92% of neonates with BA, with no statistically significant trend over time.

## Discussion

To our knowledge, this is the first large-scale analysis of the trends in diagnosis and management of obstetric and neonatal complications in DHs in Bihar, allowing a nuanced look at the continuum of care during a high-intensity nurse-mentoring program. Results suggest that diagnoses of HDP and LBW increased, while diagnoses of PPH and BA largely remained stable. Diagnoses of PPH and HDP remained below the expected population incidence. Performance of key NR steps and administration of TXA for PPH increased during the intervention period.

### Complication diagnosis

Increases were observed in the diagnoses of HDP in mothers and LBW in infants, indicating that nurse-mentoring may have contributed to these improvements. We believe the increases in observed diagnoses likely reflects real improvements in diagnosis, given that the underlying population rates is unlikely to have changed significantly over the relatively short six-month intervention period. Further, the overall rates remained below those expected in similar low resourced populations. Improvements in identification of all complications and eliminating false negative diagnosis is the critical first step towards appropriate treatment.

Nevertheless, the proportions of patients with PPH and HDP remained below expected incidences [7,8]. The overall PPH diagnosis of 3.7% was lower than the 5% suggested in the literature when PPH is determined by estimated blood loss, as was done in this study’s facilities, where resources inhibited more precise quantification [7]. At 2.1%, the proportion of patients with HDP was significantly less than the incidence of 5–10% reported in the literature [8]. The likely high number of undiagnosed PPH and HDP cases remains a concern as these are the two leading causes of maternal mortality.

The proportion of neonates with BA did not change over the course of the intervention and remained at the low end of estimated incidence [27]. Stillbirth rates in this sample were consistent with the estimated global rates of stillbirth, so it is unlikely that BA neonates were misdiagnosed as stillbirths [1]. However, it is possible that BA infants who only needed minimal stimulation to resuscitate were not recorded as asphyxiated.

Neonates with a diagnosis of LBW increased from 11% to 16% during the intervention period. While the Government of India estimates the incidence of LBW in Bihar at 9%, other studies have concluded that the true incidence is likely much higher, up to 30% [9,28]. Thus, it is likely that increases in LBW reflect improvements in identification or reporting. This could also be a consequence of increased use of digital scales. Supported by the nurse mentors, the providers felt more confident in using the digital instruments. Improved identification of LBW neonates is critical for treating the many complications of preterm birth and fetal growth restriction, such as hypothermia, hypoglycemia, poor feeding, and respiratory, cardiovascular, gastrointestinal, and neurological complications. Improvements in reporting are also helpful for tracking population incidence and addressing root causes such as maternal nutritional status and placental disorders.

We believe the greater than 50% relative increases in diagnosis of HDP in mothers and LBW in infants are clinically significant given the large population over which these increases occurred and the fact that these two diagnoses are leading causes of severe maternal and neonatal outcomes.

### Complication management

Increases were observed in the performance of initial NR steps. For NR, warming, drying, stimulating, suctioning, and administering supplemental oxygen without PPV were performed more frequently among BA infants in month 6 than in month 1 of the intervention. There remains room for improvement in the utilization of these steps as all BA infants should be warmed, dried, stimulated in the first minute after birth. Though, there was a statistically non-significant decrease over time in the use of PPV among BA infants. However, the observed rate was consistent with population estimates (3–6%) of neonates who should receive PPV [27].

Almost all PPH patients received fluids throughout the intervention, which is notable as an appropriate response to PPH given that IV fluids are not otherwise routinely given in this setting. There was a fluctuating trend in uterotonics use, with an initial decrease followed by a rise to near universal use in month 6. Field staff suggested that this could be due to improvements in emphasis on uterotonics use and improved supply over the course of the intervention period. The proportion of PPH patients receiving TXA more than doubled over the course of the intervention, during which new WHO guidance on the use of TXA in PPH came into being, supply of TXA to the hospitals increased, and data collection on TXA became more consistent [29]. We did not have data for a detailed analysis of the management of LBW neonates.

### Study strengths and limitations

Strengths of our study include its novelty of setting and scope. Specific to the AMANAT intervention, previous studies have demonstrated positive changes in obstetric and neonatal care in BEmONC facilities [22,30–32]. This study contributes evidence from the public DHs setting, to the growing body of evidence. More broadly, it adds to the literature on the changes during a nurse-mentoring program on uptake of evidence based obstetric and neonatal practices in low and middle-income CEmONC settings, by assessing a multifaceted quality improvement intervention program at a higher level of care than prior studies [17–19]. In addition, disentangling complication diagnosis from treatment enabled us to assess where gaps in care exist for specific complications.

Limitations of the study include lack of a control group, which limit our ability to attribute changes in diagnosis and treatment to the intervention. In addition, data were collected by AMANAT mentors participating in the intervention program, rather than independent clinical observers. Whilst this facilitated the large sample size by integrating data collection into routine mentoring activities, it limited the ability to suggest if the changes observed were entirely due to the change in skills of the nurse mentees. Furthermore, we did not have data on maternal and neonatal infections, which account for a large percentage of global maternal and neonatal morbidity and mortality. A key limitation in our opinion was not being able to fully assess the timeliness or appropriateness of treatment for complications without the full patient history being available in our dataset.

For the 4% of observed admissions that were referred from lower level facilities, some management steps may have been completed prior to arrival at DHs, which may have impacted what steps were indicated in the DHs. This was most salient in the case of NR, where initial warm, dry, stim steps should have been performed at the referring institution. Out dataset did not distinguish between pre- and post-partum admissions. However, the increases seen in initial NR steps were robust to dropping referred admissions. Finally, with a total of 9 maternal deaths and 10 neonatal deaths we did not have a enough sample size to analyze trends in maternal and neonatal deaths.

## Conclusions

Analysis of a large dataset of obstetric admissions in 22 DHs showed that diagnoses of HDP and LBW as well as performance of initial management steps of NR increased over the course of the AMANAT-CEMONC quality improvement initiative, suggesting the potential efficacy of a comprehensive quality improvement approach. This is the second study showing improvements in maternal and neonatal care over the course of an AMANAT intervention. However, underdiagnoses of PPH and HDP remain critical barriers to improving maternal morbidity and mortality, in this setting.

## Supporting information

S1 Table Complications by month for observed direct admissions to 22 CEMONC facilities in Bihar, India during the AMANAT intervention. P for Pearson’s chi-square test of linear trend over six months. Some patients had both preeclampsia and eclampsia during an admission, but only counted once under hypertensive disorders. PPH = Postpartum hemorrhage, PIH = Pregnancy-induced hypertension, PROM = Prelabor rupture of membranes. *Too few observations for Pearson’s chi-square test of linear trend.
(DOCX)

S2 Table Complication management by month for observed admissions to 22 CEMONC facilities in Bihar, India during the AMANAT intervention. P for chi-square test of linear trend over six months.
(DOCX)

## Abbreviations

ANM: auxiliary nurse midwife
CPR: cardiopulmonary resuscitation
FIS: Facility Information System
GNM: general nurse midwife
MMR: maternal mortality ratio
NMR: neonatal morality ratio
NR: neonatal resuscitation
NSVD: normal spontaneous vaginal delivery
PE: preeclampsia
PHC: primary health center
PPH: post-partum hemorrhage
PPV: positive pressure ventilation
UCSF: University of California San Francisco

## References

[1] GBD 2015 Child Mortality Collaborators. Global, regional, national, and selected subnational levels of stillbirths, neonatal, infant, and under-5 mortality, 1980–2015: a systematic analysis for the Global Burden of Disease Study 2015. *Lancet*. 2016;388(10053):1725–1774. 10.1016/S0140-6736(16)31575-6 27733285 PMC5224696

[2] Kassebaum NJ, Barber RM, Bhutta ZA, et al. Global, regional, and national levels of maternal mortality, 1990–2015: a systematic analysis for the Global Burden of Disease Study 2015. The Lancet. 2016;388(10053):1775–1812. 10.1016/S0140-6736(16)31470-2 27733286 PMC5224694

[3] UNICEF. About Bihar. State Info | UNICEF. http://unicef.in/StateInfo/Bihar/Introduction (Accessed March 13, 2017).

[4] World Bank Open Data | Data. https://data.worldbank.org/. Accessed February 11, 2020.

[5] State Statistics | NITI Aayog. https://niti.gov.in/state-statistics. Accessed September 10, 2019.

[6] United Nations. Goal 3: Sustainable Development Knowledge Platform. https://sustainabledevelopment.un.org/sdg3. (Accessed June 19, 2020).

[7] Deneux-Tharaux C, Bonnet MP, Tort J. [Epidemiology of post-partum haemorrhage]. J Gynecol Obstet Biol Reprod (Paris). 2014;43(10):936–950. 10.1016/j.jgyn.2014.09.023 25447386

[8] Ying W, Catov JM, Ouyang P. Hypertensive Disorders of Pregnancy and Future Maternal Cardiovascular Risk. J Am Heart Assoc. 2018; 7(17):e009382. 10.1161/JAHA.118.009382 30371154 PMC6201430

[9] Bhilwar M, Upadhyay RP, Yadav K, et al. Estimating the burden of “weighing less”: A systematic review and meta-analysis of low birth-weight in India. Natl Med J India. 2016;29(2):73–81. 27586210

[10] Campbell OMR, Graham WJ, Lancet Maternal Survival Series steering group. Strategies for reducing maternal mortality: getting on with what works. Lancet. 2006;368(9543):1284–1299. 10.1016/S0140-6736(06)69381-1 17027735

[11] Singh SK, Kaur R, Gupta M, Kumar R. Impact of National Rural Health Mission on perinatal mortality in rural India. Indian Pediatr 2012; 49: 136–8. 10.1007/s13312-012-0022-8 21992866

[12] Souza JP, Gülmezoglu AM, Vogel J, et al. Moving beyond essential interventions for reduction of maternal mortality (the WHO Multicountry Survey on Maternal and Newborn Health): a cross-sectional study. Lancet. 2013;381(9879):1747–1755. 10.1016/S0140-6736(13)60686-8 23683641

[13] Lawn JE, Lee AC, Kinney M, et al. Two million intrapartum-related stillbirths and neonatal deaths: Where, why, and what can be done? Int J Gynaecol Obste. 2009; 107, Supplement: S5–19. 10.1016/j.ijgo.2009.07.016 19815202

[14] Paxton A, Maine D, Freedman L, Fry D, Lobis S. The evidence for emergency obstetric care. Int J Gynaecol Obste. 2005; 88: 181–93. 10.1016/j.ijgo.2004.11.026 15694106

[15] Siassakos D, Crofts J, Winter C, Weiner C, Draycott T. The active components of effective training in obstetric emergencies. BJOG. 2009; 116(8): 1028–32. 10.1111/j.1471-0528.2009.02178.x 19438497

[16] Bergh AM, Baloyi S, Pattinson RC. What is the impact of multi-professional emergency obstetric and neonatal care training? Best Pract Res Clin Obstet Gynaecol. 2015; 29: 1028–43. 10.1016/j.bpobgyn.2015.03.017 25937554

[17] Semrau KEA, Hirschhorn LR, Delaney MM, et al. Outcomes of a Coaching-Based WHO Safe Childbirth Checklist Program in India. N Engl J Med. 2017;377(24):2313–2324. 10.1056/NEJMoa1701075 29236628 PMC5672590

[18] Goudar SS, Somannavar MS, Clark R, et al. Stillbirth and Newborn Mortality in India After Helping Babies Breathe Training. *Pediatrics*. 2013;131(2):e344–e352. 10.1542/peds.2012-2112 23339215

[19] Msemo G, Massawe A, Mmbando D, et al. Newborn Mortality and Fresh Stillbirth Rates in Tanzania After Helping Babies Breathe Training. *Pediatrics*. 2013;131(2):e353–e360. 10.1542/peds.2012-1795 23339223

[20] Walker DM, Holme F, Zelek ST, et al. A process evaluation of PRONTO simulation training for obstetric and neonatal emergency response teams in Guatemala. *BMC Med Educ* 2015; 15. 10.1186/s12909-015-0401-7 26206373 PMC4513701

[21] Walker DM, Cohen SR, Fritz J, et al. Impact Evaluation of PRONTO Mexico: A Simulation-Based Program in Obstetric and Neonatal Emergencies and Team Training. *Simulation in Healthcare*: *The Journal of the Society for Simulation in Healthcare* 2016; 11: 1–9. 10.1097/SIH.0000000000000106 PMC5367503 26312613

[22] Ghosh R, Spindler H, Morgan MC, et al. Diagnosis and management of postpartum hemorrhage and intrapartum asphyxia in a quality improvement initiative using nurse-mentoring and simulation in Bihar, India. PLoS One. 2019 7 5;14(7):e0216654. 10.1371/journal.pone.0216654 31276503 PMC6611567

[23] Bihar Health Management Information System. http://statehealthsocietybihar.org/hmis_report.html. Accessed March 26, 2020.

[24] Davidoff F, Batalden P, Stevens D, Ogrinc G, Mooney SE; SQUIRE development group. Publication guidelines for quality improvement studies in health care: evolution of the SQUIRE project. BMJ. 2009; 338:a3152. 10.1136/bmj.a3152 19153129 PMC2769030

[25] Stead K, Kumar S, Schultz TJ, et al. Teams communicating through STEPPS. Med J Aust. 2009;190(S11):S128–S132. 10.5694/j.1326-5377.2009.tb02619.x 19485861

[26] WHO recommendations for prevention and treatment of pre-eclampsia and eclampsia. Geneva, Switzerland: World Health Organization; 2011. 23741776

[27] Lee AC, Cousens S, Wall SN, et al. Neonatal resuscitation and immediate newborn assessment and stimulation for the prevention of neonatal deaths: a systematic review, meta-analysis and Delphi estimation of mortality effect. BMC Public Health. 2011;11(3):S12. 10.1186/1471-2458-11-S3-S12 21501429 PMC3231885

[28] NITI Aayog. Healthy States Progressive India: Report on the Ranks of States and Union Territories. Published online June 2019. http://social.niti.gov.in/uploads/sample/health_index_report.pdf. (Accessed June 16, 2020).

[29] WHO Recommendation on Tranexamic Acid for the Treatment of Postpartum Haemorrhage. Geneva: World Health Organization; 2017. http://www.ncbi.nlm.nih.gov/books/NBK493081/. (Accessed January 24, 2020). 29630190

[30] Das A, Nawal D, Singh MK, et al. Impact of a Nursing Skill-Improvement Intervention on Newborn-Specific Delivery Practices: An Experience from Bihar, India. *Birth* 2016; 43: 328–35. 10.1111/birt.12239 27321470

[31] Vail B, Morgan MC, Spindler H, Christmas A, Cohen SR, Walker DM. The power of practice: simulation training improving the quality of neonatal resuscitation skills in Bihar, India. *BMC Pediatr*. 2018;18(1):291. 10.1186/s12887-018-1254-0 30176831 PMC6122678

[32] Das A, Nawal D, Singh MK, et al. Evaluation of the mobile nurse training (MNT) intervention—a step towards improvement in intrapartum practices in Bihar, India. *BMC Pregnancy Childbirth*. 2017;17. 10.1186/s12884-017-1452-z 28835213 PMC5569501

