# Supplementary material for: Increases in diagnosis and management of obstetric and neonatal complications in district hospitals during a high intensity nurse-mentoring program in Bihar, India
Source: PLoS One. 2021 Mar 18;16(3):e0247260. doi: 10.1371/journal.pone.0247260 (PMC7971704; doi:10.1371/journal.pone.0247260)
Supplement: S1 Table — P for Pearson’s chi-square test of linear trend over six months. Some patients had both preeclampsia and eclampsia during an admission, but only counted once under hypertensive disorders. PPH = Postpartum hemorrhage, PIH = Pregnancy-induced hypertension, PROM = Prelabor rupture of membranes. *Too few observations for Pearson’s chi-square test of linear trend. (DOCX) [file pone.0247260.s001.docx]

**Supplementary information**

**Table S1:** Complications by month for observed direct admissions to 22 CEMONC facilities in Bihar, India during the AMANAT intervention


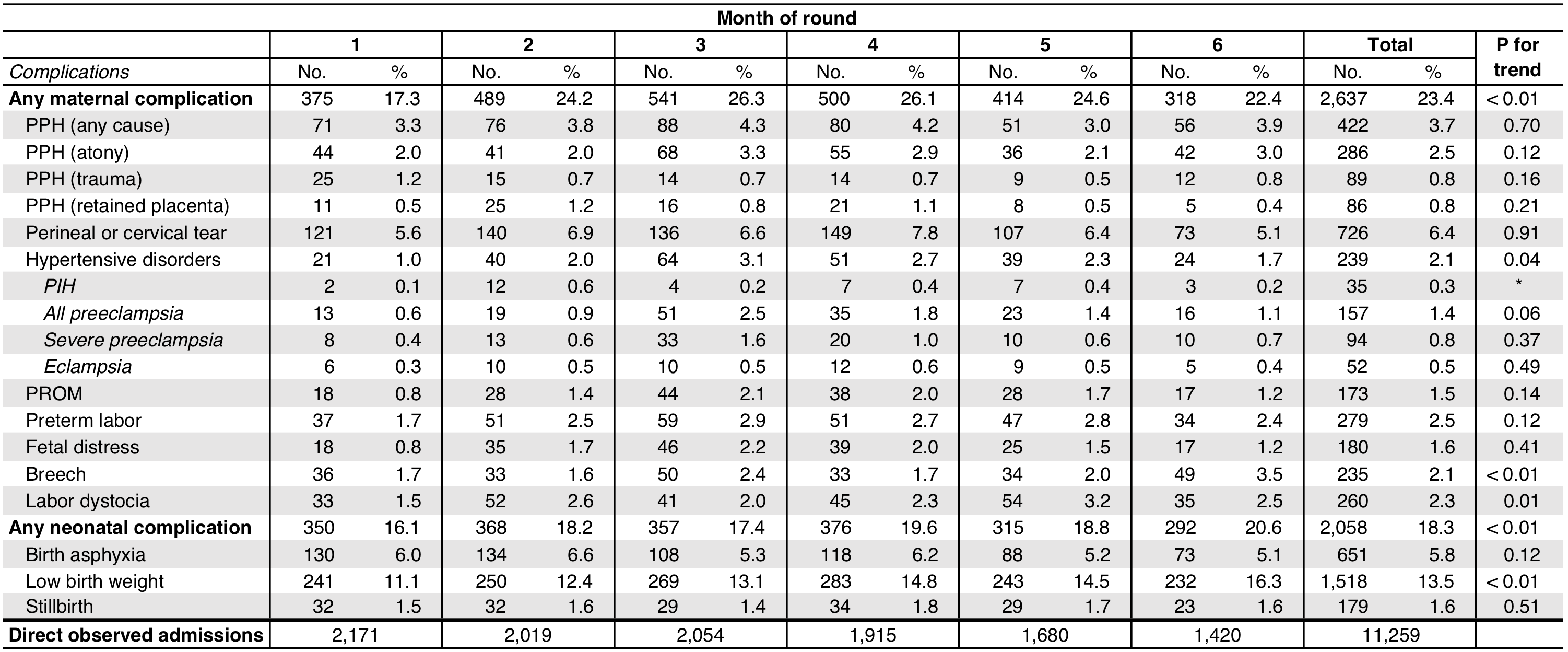


P for Pearson’s chi-square test of linear trend over six months

Some patients had both preeclampsia and eclampsia during an admission, but only counted once under hypertensive disorders

PPH = Postpartum hemorrhage, PIH = Pregnancy-induced hypertension, PROM = Prelabor rupture of membranes

*Too few observations for Pearson’s chi-square test of linear trend
