# Supplementary material for: Increases in diagnosis and management of obstetric and neonatal complications in district hospitals during a high intensity nurse-mentoring program in Bihar, India
Source: PLoS One. 2021 Mar 18;16(3):e0247260. doi: 10.1371/journal.pone.0247260 (PMC7971704; doi:10.1371/journal.pone.0247260)
Supplement: S2 Table — P for chi-square test of linear trend over six months. (DOCX) [file pone.0247260.s002.docx]

**Table S2:** Complication management by month for observed admissions to 22 CEMONC facilities in Bihar, India during the AMANAT intervention

**
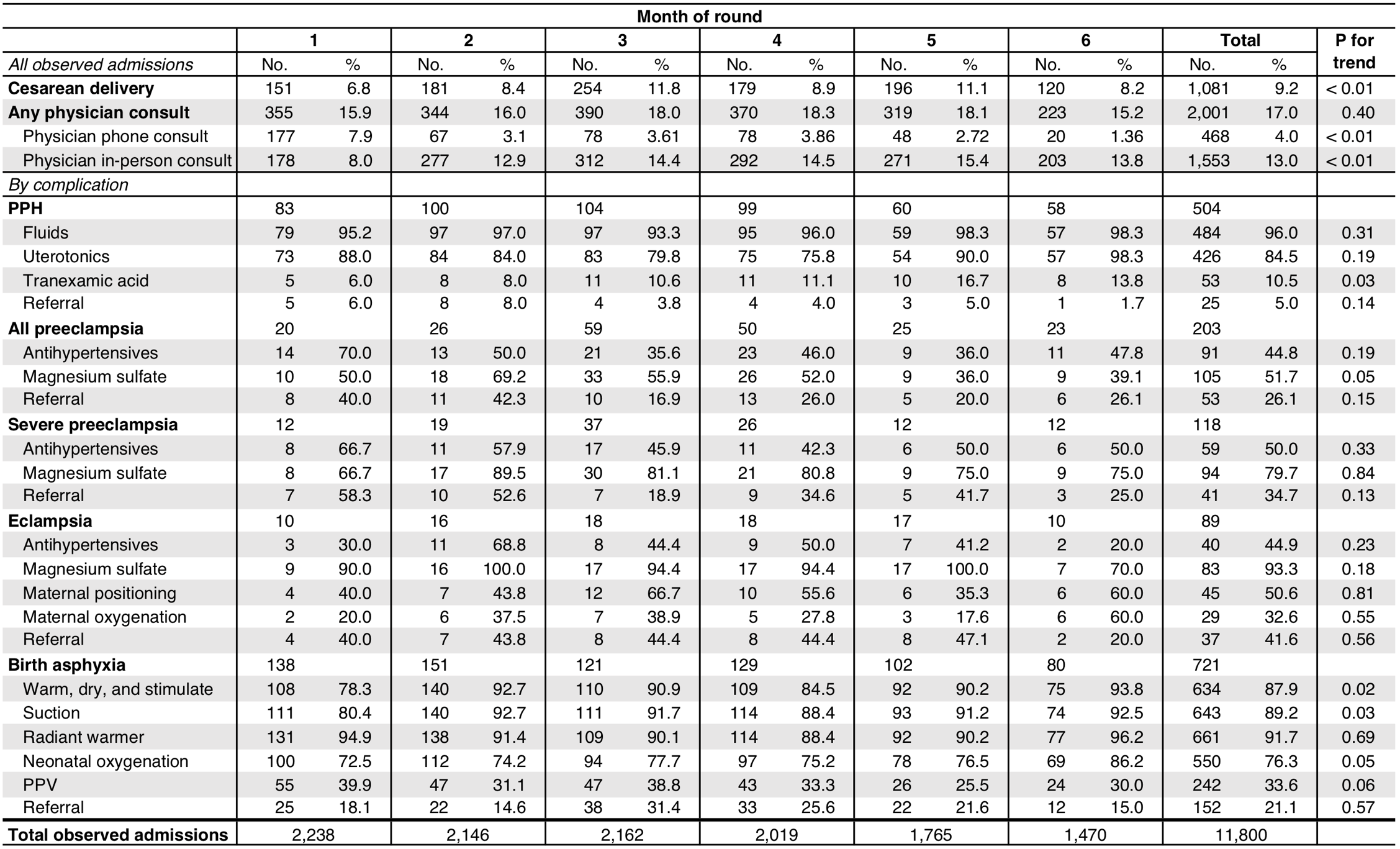
**

P for chi-square test of linear trend over six months
